# Association of vitamin D deficiency, season of the year, and latent tuberculosis infection among household contacts

**DOI:** 10.1371/journal.pone.0175400

**Published:** 2017-04-12

**Authors:** María Elvira Balcells, Patricia García, Camila Tiznado, Luis Villarroel, Natalia Scioscia, Camila Carvajal, Francesca Zegna-Ratá, Mariluz Hernández, Paulina Meza, Luis F. González, Carlos Peña, Rodrigo Naves

**Affiliations:** 1 Departamento de Enfermedades Infecciosas del Adulto, Escuela de Medicina, Pontificia Universidad Católica de Chile, Santiago, Chile; 2 Departamento de Laboratorios Clínicos, Escuela de Medicina, Pontificia Universidad Católica de Chile, Santiago, Chile; 3 Departamento de Salud Pública, Escuela de Medicina, Pontificia Universidad Católica de Chile, Santiago, Chile; 4 Programa de Microbiología y Micología, Facultad de Medicina, Universidad de Chile, Santiago, Chile; 5 Instituto de Ciencias Biomédicas, Facultad de Medicina, Universidad de Chile, Santiago, Chile; 6 Servicio de Respiratorio, Hospital San Borja Arriarán, Santiago, Chile; Rutgers Biomedical and Health Sciences, UNITED STATES

## Abstract

**Objectives:**

Vitamin D (VD) enhances the immune response against *Mycobacterium tuberculosis in vitro*, and VD deficiency has been described in patients with active tuberculosis (TB). However, the role of hypovitaminosis D in the pathogenesis of early TB infection acquisition is unclear. We aimed to evaluate the association of VD deficiency, season of the year, and latent TB infection in household contacts (HHC), given that this is a potentially modifiable condition often related to nutritional deficiencies and lack of sun exposure.

**Methods:**

We prospectively enrolled new pulmonary TB cases (n = 107) and their HHC (n = 144) over a 2-year period in Santiago, Chile. We compared plasma 25-hydroxycholecalciferol (25OHD) levels and examined the influence of season, ethnic background, living conditions, and country of origin.

**Results:**

Over 77% of TB cases and 62.6% of HHC had VD deficiency (<20 ng/ml). Median 25OHD concentration was significantly lower in TB cases than in HHC (11.7 vs. 18.2 ng/ml, p<0.0001). Migrants HHC had lower 25OHD levels than non-migrants (14.6 vs. 19.0 ng/ml, p = 0.026), and a trend towards a higher burden of latent TB infection (52.9% vs. 35.2%, p = 0.066). Multivariate analysis found VD deficiency in HHC was strongly associated with being sampled in winter/spring (_ad_OR 25.68, 95%CI 7.35–89.7), corresponding to the seasons with lowest solar radiation exposure. Spring enrollment–compared with other seasons–was the chief risk factor for latent TB infection in HHC (_ad_OR 3.14, 95%CI 1.28–7.69).

**Conclusions:**

Hypovitaminosis D was highly prevalent in TB cases and also in HHC. A marked seasonality was found for both VD levels and latent TB in HHC, with winter being the season with lowest VD levels and spring the season with the highest risk of latent TB infection.

## Introduction

Tuberculosis (TB) is still currently a serious public health concern, with more than 10.4 million people worldwide having developed the disease in 2015 [1]. However, with global progress and development, a growing number of countries are aiming to advance towards TB elimination in the next decades. Global strategy for TB decline requires country adaptation and prioritization, according to the local epidemiology and available TB control resources [2]. In Chile, as well as in many low TB prevalence countries, reaching that goal will require increasing efforts to address TB transmission among groups of previously healthy individuals that share social risk factors such as imprisonment, poverty, homelessness and being an immigrant [3]. In addition, tackling latent TB, particularly among recently exposed household contacts, will be a key priority as the large reservoir of asymptomatic infected subjects threatens TB elimination [2].

Undernutrition has long been recognized as a major driver of TB epidemics [4,5]. Among specific nutritional factors that may be in deficit without overt malnutrition, vitamin D (VD) deficiency has been associated with active pulmonary and extrapulmonary TB in different geographical areas [6–9]. Besides its major role in bone metabolism, VD has been known to be important for protecting against infection. Several studies have revealed that 1,25-dihydroxyvitamin D (1,25(OH)D3), which is the active form of VD, plays a key protective role against mycobacteria, boosting the innate immune system and influencing adaptive immunity by modulating antigen presentation [10,11].

A particularity of VD is that it is barely available in the common diet, with a limited amount coming from sources such as oily fish. VD requirements are mainly fulfilled from endogenous synthesis after exposure of 7-dehydrocholesterol in the skin to solar ultraviolet radiation [12]. Low ultraviolet B (UVB) exposure and modern lifestyle are among the factors that contribute to the 30−50% of adults and children worldwide who are at risk of VD deficiency [12]. Hypovitaminosis D is usually accentuated in seasons of low UVB exposure, and in the city of Santiago (central Chile, latitude 33° S), studies show that VD deficiency can reach as high as 60% in postmenopausal women [13].

Unlike other respiratory infections, active TB notifications display clear seasonality in latitudes with distinct summer and winter seasons, and worldwide, TB diagnoses generally increase in summer [14,15]. Among factors that may explain this phenomenon it has been hypothesized that a higher susceptibility to progression to active TB may occur among subjects under VD deficiency conditions arising as a consequence of decreased ambient exposure to UVB light in winter [16].

What has been scarcely explored thus far is the role of VD deficiency and seasonality in the susceptibility of acquiring latent TB infection (LTBI) in exposed contacts. The rationale being that if VD boosts innate immune response to mycobacteria, VD deficiency occurring in low radiation seasons could impair the clearance of an early TB infection. Therefore in the present study, we aimed to evaluate the association between season, VD levels, active pulmonary TB, and LTBI in household contacts (HHC).

## Methods

### Study design

In a prospective, cross-sectional study, conducted between August 2013 and December 2015, all new pulmonary TB patients, from 19 outpatient clinics and 3 local hospitals belonging to central Santiago health services area, and their HHC were invited to participate. Inclusion criteria for active TB cases were any patient (≥15 years old) with positive sputum smear microscopy, in addition to radiological evidence of lung involvement. Exclusion criteria for TB cases were patients having already initiated TB treatment (>7 days), subjects imprisoned at diagnosis, and subjects with a non-tuberculous mycobacterium identified in final sputum culture. Patients with active TB and HIV infection were only excluded from VD and cytokine analysis.

Inclusion criteria for HHC were subjects ≥15 years old that had resided in the household for at least 7 consecutive days during the 3 months prior to the diagnosis of TB in index case [17]. HHC were only enrolled 8 to 10 weeks after TB was diagnosed in active TB cases, and all HHC were clinically evaluated to rule out active TB by symptoms screening and a chest X-ray. We excluded from the analysis HHC with any symptom or radiological sign of active TB, any ongoing vitamin supplement intake, pregnant women, known HIV positive contacts, and those reporting having had past TB. Additionally, we included a small control group (non-HHC) with subjects chosen among healthy volunteers with no known TB risk factors (not migrants, no past TB, not working in health care or correctional facilities, no known TB contact) and having a negative latent TB test at enrollment.

General epidemiological data (age, sex, ethnicity, country of origin, time since country arrival, employment, living conditions) and known TB risk factors (comorbidities including HIV, diabetes, current medications, smoking and alcohol consumption, BCG status, duration of TB exposure and intensity of smear of index cases) were recorded. Indigenous origin was determined by self-reporting or if the patient’s last name was of indigenous origin. Crowding index was determined for each household unit as the number of individuals living in the house divided by the number of bedrooms [18]. Cough duration in index case was defined as the number of days of cough before diagnosis was established by a positive acid-fast smear in sputum.

### Laboratory assessment

Bacterial confirmation of pulmonary TB cases was carried out by sputum solid and liquid mycobacterial culture (MGIT). *M*. *tuberculosis* complex species were confirmed for all strains by a specific PCR for gyrase B (gyrB) with primers described by Kasai et al [19].

Blood samples (20 ml per subject) were collected at the time of diagnosis in active TB cases and 8–10 weeks later in contacts. LTBI was investigated with QuantiFERON TB Gold ® test (QFT) (Cellestis), and remaining plasma and serum were stored at -80°C. Plasma levels of 25-hydroxyvitamin D (25OHD) were determined by liquid chromatography—mass spectrometry (LC-MS/MS) at the Laboratorio Clínico from Pontificia Universidad Católica de Chile. This is a reference and certified laboratory, which participates in DEQAS (The Vitamin D External Quality Assessment Scheme) program. A 25OHD plasma concentration <20 ng/mL (50 nmol/L) was considered an indication of VD deficiency for present analysis [12]. Plasma levels of tumor necrosis factor α (TNF-α) and interleukin 6 (IL-6) were determined by Multiplex immunoassay (HCYTOMAG, MILLIPORE). High-sensitivity C-reactive protein (CRP) was determined by nephelometry (BN ProSpec® System SIEMENS).

### Data analysis

Continuous data were summarized with median and ranges and compared using the non-parametric Mann–Whitney U test or Kruskal-Wallis for multiple comparisons. Categorical responses were expressed as a percentage, and comparisons were made using Pearson’s χ2 test (or Fisher’s exact test if appropriate). To evaluate association between 25OHD concentration and other inflammatory biomarkers we used Spearman´s correlation. Univariable and multivariable analysis of risk factors associated with LTBI acquisition in contacts were assessed using logistic regression and reported as crude ORs and adjusted ORs (95% CIs). To evaluate associations in 25OHD concentration and hypovitaminosis D among HHC, we mutually adjusted for age, sex, season at recruitment (winter/spring vs. summer/autumn), migrancy, crowding index and smoking. All analyses were done with SPSS statistical software for Windows, Version 17.0 (Chicago: SPSS Inc.) and figures with GraphPad Prism version 7.0 for Windows (GraphPad Software, La Jolla California USA). All tests were two tailed; p values ≤0.05 were considered significant.

### Ethics and consent to participate

Ethical approval was obtained from the Ethics Committee of the Pontificia Universidad Católica de Chile’s Faculty of Medicine and from the Servicio de Salud Metropolitano Central Ethics Committee. All eligible patients provided written informed consent, according to institutional requirements. We obtained consent from parents and written assent from minors. All contacts having a positive QFT result were referred to the local TB program provider to evaluate the need of chemoprophylaxis.

## Results

### General characteristics of enrolled subjects

During this period, 107 acid-fast smear positive cases of pulmonary TB, their 146 HHC and 32 non-HHC were enrolled. Two HHC were excluded after finding co-prevalent active TB on screening and 5 HHC reporting having had past TB were excluded from further analysis. Demographic and clinical characteristics of enrolled subjects are described in detail in **Table 1**. Subjects diagnosed with active TB were predominantly male (65.4%), with a median age of 37 years old and HIV co-infection was detected in 11% of cases. A total of 29% of all TB cases corresponded to cases diagnosed in migrants from high TB endemic countries (>95% from neighbor countries Bolivia and Peru). Median time since arrival to Chile in migrants was 36 months (range: 2–360 months), although 63% had travelled again to their country of origin at least one time after initial arrival.

**Table 1:** Clinical and demographic characteristics of tuberculosis (TB) cases, household contacts (All HHC) and non-household contacts control group (Non-HHC) at enrollment.

	Active pulmonary TB cases (N = 107)	All HHC (N = 144)	Non-HHC (N = 31)	*P* value (HHC vs. non-HHC)
Median age in years (IQR)	37 (29–53)	37 (26–52)	32 (26–48)	0.52
Male sex–Number (%)	70 (65.4%)	64 (44.4%)	18 (58.1%)	0.233
HIV positive- Number (%)	12 (11.2%)	ND	ND	…
Previous/past TB- Number (%)	20 (18.7%)	5 (3.5%)	0 (0%)	…
Diabetes mellitus -Number (%)	11 (10.3%)	7 (4.9%)	1 (3.2%)	0.999
Smoking–Number (%)	37 (34.6%)	61 (42.4%)	9 (29%)	0.225
Median Crowding Index (IQR)	2.0 (1.3–2.3)	1.87 (1.5–2.33)	1.0 (1–1)	<0.0001
Homeless—Number (%)	10 (9.3%)	NA	NA	…
Ethnic background - Non-indigenous (caucassians and mestizo)- Number (%) - Indigenous (natives*)—Number (%) - African-americans- Number (%)	- 86 (80.4%) - 19 (17.7%) - 2 (1.9%)	- 134 (93%) - 9 (6.3%) - 1 (0.7%)	- 31 (100%) - 0 (0%) - 0 (0%)	0.26 0.37 …
Migrants from country with TB rate >100 per 100,000 population- Number (%)	31 (29%)	37 (25.7%)	0 (0%)	…
Ever employed or stayed in a health care or a correctional facility. Number (%)	2 (1.9%)	4 (2.8%)	0 (0%)	0.999
Works or study outside the house-Number (%)	61 (57%)	98 (68%)	28 (90.3%)	0.014
BCG scar present- Number (%)	88 (82.2%)	131 (91%)	31 (100%)	0.128
QFT-G result positive**- Number (%)	ND	55 (39.6%)	0 (0%)	…

QFT-G = Quantiferon TB Gold Test In Tube; ND: Not determined; NA: not applicable; Crowding index: n° individuals living in the household/ n° bedrooms

(*) Indigenous origin included Mapuches and Aymaras

(**) excluding 5 HHC reporting past TB

Among enrolled HHC, we detected a very high prevalence of LTBI, with positive QFT test results in 39.6% (55/139). One of the non-HHC subjects had positive QFT test result and was excluded from further analysis. Non-HHC differed from HHC in that a higher proportion of subjects worked or studied outside the household (90.3% vs. 68%, p = 0.01), and their household crowding index was lower (1.05 vs. 1.95, p<0.0001).

### Factors associated with latent TB infection in contacts

Positivity of latent TB test among HHC was higher in male contacts (51.7% vs. 30.4% in females, p = 0.011); in those exposed to index cases with higher sputum smear count (50% vs 31.6% among subjects exposed to lower intensity smears, p = 0.028); and in those working or studying outside the household (46.3% vs. 25%, p = 0.025). Also, HHC sampled in spring had a higher probability of having LTBI than HHC sampled in other seasons (62.5% vs. 32.7%, p = 0.004). Multivariate analysis found LTBI in contacts was only strongly associated with being tested in spring (OR 3.14, 95%CI 1.28–7.69) (**Table 2**).

**Table 2:** Regression Analysis of associations with latent TB infection among household contacts (n = 139).

	Univariate Analysis (binomial)				Multivariate logistic regression (binomial)	
	QFT(+) HHC (n = 55)	QFT(-) HHC (n = 84)	OR (95% CI)	*P* value	Adjusted OR (95% CI)	*P* value
**Age; years (IQR)**	37 (25–53)	36 (25–52)	0.99 (0.97–1.02)	0.715	1.01 (0.99–1.03)	0.428
**Sex (male)-number (%)**	31 (56.4%)	29 (33.3%)	2.45 (1.24–5.04)	**0.011**	2.2 (0.97–4.99)	0.06
**Season at enrollment:—Spring: number (%)**	20 (36.4%)	12 (14.3%)	3. 43 (1.47–7.54)	**0.004**	**3.14 (1.28–7.69)**	**0.012**
**Median Household Crowding Index (IQR)**	2 (1.5–2.4)	1.87 (1.45–2.3)	1.41 (0.89–2.24)	0.14	…	…
Migrants** vs. non-migrants	18 (32.7%)	16 (19%)	2.07 (0.93–4.33)	0.066	2.08 (0.89–4.87)	0.09
**Works or studies outside the house- Number (%)**	44 (80%)	51 (60.7%)	2.59 (1.2–5.4)	**0.025**	2.19 (0.83–5.78)	0.112
**Indigenous origin, Number (%)**	5 (6.2%)	4 (4.8%)	2.00 (0.56–6.73)	0.319	…	…
Median duration of exposure(*) in days (IQR)	66 (32–147)	74 (32–165)	0.99 (0.99–1.00)	0.72	…	…
**Higher intensity of acid fast smear in index case (+++)- Number (%)**	30 (54.5%)	30 (35.7%)	2.16 (1.1–4.4)	**0.028**	2 (0.93–4.29)	0.077
**BCG scar present- Number (%)**	48 (87.3%)	78 (92.9%)	0.53 (0.16–1.54)	0.372	…	…
**Diabetes mellitus-Number (%)**	2 (3.6%)	5 (5.9%)	0.59 (0.12–2.96)	0.703	…	…
**Smoking-Number (%)**	21 (38.2%)	36 (42.9%)	0.82 (0.4–1.63)	0.602	…	…
**Median (IQR) plasma VD (ng/ml)**	17.3 (11.8–23.5)	18.25 (12.1–22.7)	0.87 (0.95–1.04)	0.611	…	…
**Prevalence of VD deficiency (VD<20 ng/ml)-Number (%)**	35 (63.6%)	52 (61.9%)	1.08 (0.53–2.16)	0.837	…	…

* Cough duration in Index Case before TB diagnosis was made.

** migrants from countries with recent TB rates >100 per 100.000 population. QFT: Quantiferon TB Gold test, HHC: household contacts

### Levels of 25OHD sufficiency in subjects with active pulmonary TB, HHC and non-HHC

**Fig 1:**
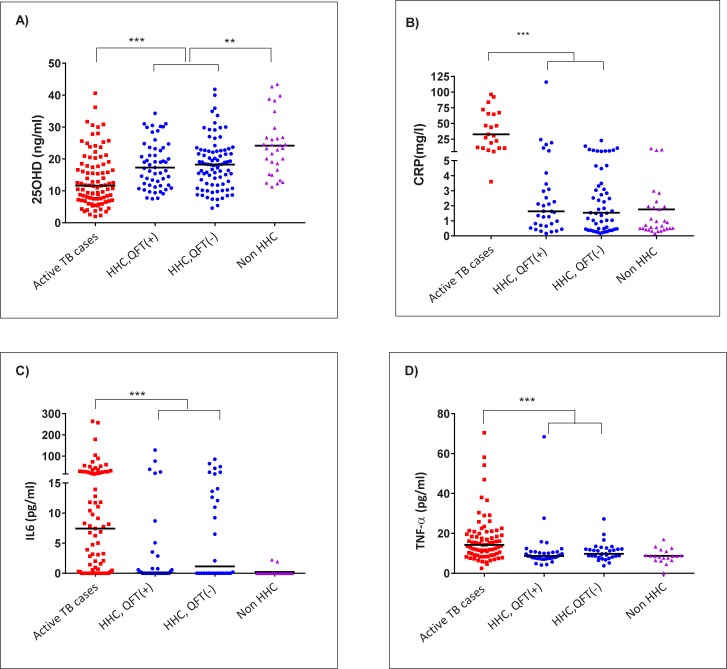
Plasma levels of 25OHD and inflammatory parameters in active pulmonary TB cases, household contacts (HHC) and non-HHC subjects. **A)** 25OHD levels in active pulmonary TB cases (n = 92), household contacts (HHC) with latent TB infection (QFT(+), n = 55), HHC without latent TB infection (QFT(-), n = 84) and non-HHC subjects (n = 31). **B)** Ultrasensitive C-reactive protein (CRP) in active pulmonary TB cases (n = 23), HHC with latent TB infection (QFT(+), n = 33), HHC without latent TB infection (QFT(-), n = 53) and non-HHC subjects (n = 31). **C)** Interleukin 6 (IL-6) in active pulmonary TB cases (n = 79), HHC with latent TB infection (QFT(+), n = 36), HHC without latent TB infection (QFT(-), n = 32) and non-HHC subjects (n = 19). **D**) TNF-α in active pulmonary TB cases (n = 79), HHC with latent TB infection (QFT(+), n = 36), HHC without latent TB infection (QFT(-), n = 32) and non-HHC subjects (n = 19). (*** = p< 0.001, ** = p<0.01, * = p<0.05). Horizontal line represents the median of each subject group.

Plasma concentration of 25OHD was available for 92 TB cases (after exclusion of 12 HIV positive subjects, 2 subjects with insufficient blood sampling and 1 subject taking vitamins), for 139 HHC (5 subjects with past TB were excluded) and for all 31 non-HHC. Both individuals with active TB and HHC had low levels of 25OHD, although hypovitaminosis D was more profound among the former (median levels 11.7 vs. 18.2 ng/ml, respectively, p<0.0001). In turn, median VD levels were lower in HHC than in non-HHC (18.2 vs. 23.4 ng/ml, respectively, p = 0.001) (**Fig 1A**). In total, 77% of patients with active TB were in the range of VD deficiency (<20 ng/ml) and 42% in the range of severe VD deficiency (<10 ng/ml). With respect to HHC, 62.6% had VD deficiency and only 33.3% of the non-HHC subjects.

In addition, significantly lower levels of 25OHD were found among all migrant HHC compared to non-migrant HHC (14.6 vs 19 ng/ml, p = 0.026). This difference was not evident between migrant and non-migrant active TB cases in which hypovitaminosis D was widespread (median 25OHD levels 12.5 vs. 10.5 ng/ml, respectively, p = NS).

Given that age, sex, season of the year, migrancy status, household crowding index (as a correlate of socioeconomic status) and smoking were all covariables potentially associated with 25OHD status, we constructed a linear regression analysis. Season of enrollment was particularly relevant in determining VD status in HHC, with a median 25OHD of 14.15 ng/ml in winter/spring vs. 23.2 ng/ml in summer/autumn (p<0.0001). In the multivariable analysis, season of sampling (winter/spring vs summer/autumn) was the only factor strongly associated with lower 25OHD levels (p<0.0001) as well as with VD deficiency in HHC (OR 25.68, 95% CI 7.35–89.7) (**Table 3**).

**Table 3:** Regression Analysis of associations with Vitamin D levels among all TB household contacts (n = 139).

	25OHD plasma concentrations (ng/ml), lineal regression				Vitamin D deficiency (< 20 ng/ml), binomial regression			
	Univariate Regression		Multiple Regression		Univariate Regression		Multiple Regression	
	Coefficient β (95% CI)	*P* value	Coefficient β (95% CI)	*P* value	OR (95% CI)	*P* value	Adjusted OR (95% CI)	*P* value
**Sex (male)**	-2.3 (-4.87–0.27)	0.08	-1.21 (-3.55–1.13)	0.308	1.55 (0.77–3.13)	0.224	0.98 (0.32–3.06)	0.98
**Age; years**	-0.01 (-0.09–0.07)	0.805	-0.05 (-0.12–0.02)	0.159	0.99 (0.97–1.01)	0.605	1.01 (0.97–1.04)	0.643
**Season** (Winter/Spring vs. Summer/Autumn)	-9.52 (-11.63–-7.41)	**<0.0001**	-8.07 (-10.8–-5.4)	**<0.0001**	23.29 (9.45–57.4)	**<0.0001**	25.68 (7.35–89.67)	**<0.0001**
**Household Crowding Index**	1.94 (0.39–3.48)	**0.015**	0.52 (-0.84–1.87)	0.452	0.59 (0.37–0.97)	**0.037**	0.84 (0.39–1.85)	0.673
**Migrant contacts vs. non-migrant**	-3.49 (-6.43–-0.56)	**0.002**	-2.65 (-5.91–0.6)	0.109	1.93 (0.82–4.53)	0.133	…	…
**Indigenous origin**	-0.11 (-5.34–5.13)	0.968	…	…	1.21 (0.29–5.06)	0.794	…	…
**Smoking**	3.59 (1.04–6.13)	**0.006**	2.03 (-0.61–4.66)	0.13	0.43 (0.21–0.87)	**0.018**	0.36 (0.11–1.14)	0.083
**Works or studies outside the house**	-2.65 (-5.38–0.09)	0.058	…	**…**	1.64 (0.79–3.41)	0.184	…	…

**Fig 2:**
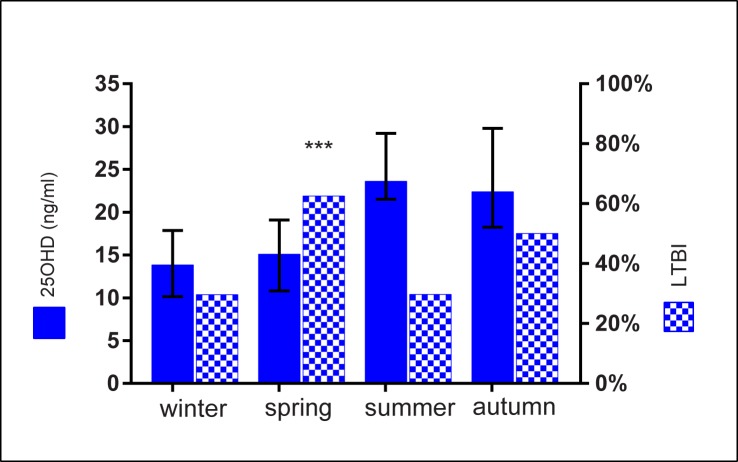
Plasma levels of 25OHD in household contacts (HHC) (n = 139) and proportion of HHC with latent TB infection (LTBI) in each season. Median value with IQR is shown. The proportion of LTBI found in HHC was higher in spring (62.5%) than in other seasons (32.7%). (*** p = 0.004).

As mentioned above, spring was the season where the highest proportion of HHC investigated had LTBI (62.5% vs. 32.7% for other seasons combined, p = 0.004) (**Fig 2**). Given that our HHC were tested for LTBI 8–10 weeks after last exposure, this finding suggests that HHC having being exposed to active TB during winter–when VD is at its lowest level–had a higher probability of acquiring LTBI than HHC exposed in other seasons of the year.

### Other inflammatory biomarkers, vitamin D, and latent TB infection

As expected, compared with HHC, active TB cases had significantly higher median plasma levels of CRP (32.7 vs. 1.6 ng/ml, p<0.0001), IL-6 (7.44 vs. 0 pg/ml, p = 0.0005) and TNF-α (14.19 vs. 9.15 pg/ml, p<0.0001) (**Fig 1B–1D**). However, we did not found a significant correlation between 25OHD levels in TB cases and any of the inflammatory biomarkers CRP (r_s_ = 0.01, p = 0.91), IL-6 (r_s_ = -0.067, p = 0.55) or TNF- α (r_s_ = -0.15, p = 0.17).

In HHC, median concentration of circulating inflammatory biomarkers did not differ between latent TB infected and uninfected subjects (**Fig 1B–1D**) and there was no significant correlation between 25OHD and CRP (r_s_ = 0.09, p = 0.62), IL-6 (r_s_ = -0.06, p = 0.73) or TNF- α (r_s_ = 0.08, p = 0.63) concentration.

## Discussion

**Fig 3:**
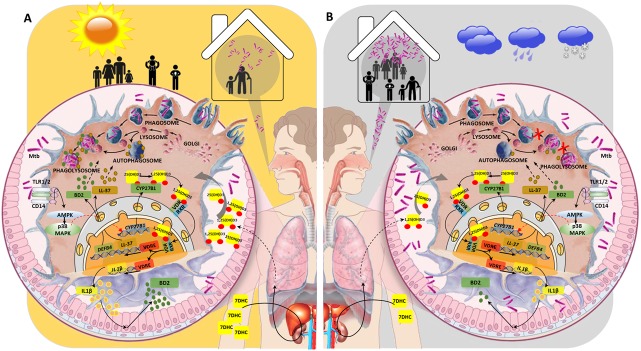
A schematic model for the impact of the seasonality and levels of Vitamin D (VD) metabolites on the risk of being infected with *M*. *tuberculosis* and the protective mechanisms of the immune response. (**A**) During spring/summer season, sunny days favor outdoor activities, decrease close contact with people with TB and increase sun light exposure promoting enhanced skin production of 7-dehydrocholesterol (7DHC), the precursor of active VD, and the synthesis of D3 metabolites first in liver and then in kidney. The main production of 1,25-dihydroxyvitamin D3 (1,25(OH)D3) occurs in the kidney but it can also be produced by inflammatory cells during an immune response to infections [10,11,23]. Alveolar macrophages recognize molecules associated with *M*. *tuberculosis*, such as the mycobacterial lipoprotein LpqH, through Toll-like receptors (TLRs) such as TLR2/1 and the co-receptor CD14 expressed on their cell surface [20,24]. Engagement of these receptors induce a cell signaling pathway that include AMPK and p38 MAPK activation, which leads to upregulation of CYP27B1 hydroxylase and the conversion of 25OHD into 1,25(OH)D3 [24]. Given that immune cells can also express the VD receptor (VDR), 1,25(OH)D3 binds to heterodimer formed between the VDR and the retinoid X receptor (RXR) and translocate into the nucleus where this transcription factor complex specifically recognizes DNA sequences named VD response elements (VDRE) leading to the production of antimicrobial peptides, such as LL-37 (cathelicidin) and ß-defensin 2 (BD2) [24]. Cathelicidin induced by 1,25(OH)D3 drives the elimination of engulfed mycobacteria by promoting fusion of autophagosomes containing mycobacteria with lysosomes [24,25]. *M*. *tuberculosis* infection and 1,25(OH)D3 induce IL-1ß gene expression, which binds via IL-1ß receptor to epithelial alveolar cells to promote the expression of BD2. The release of BD2 along with 1,25(OH)D3 contributes to control mycobacterial proliferation in the macrophage [26]. (**B**) During autumn/winter season, cold, cloudy and rainy days promote indoor lifestyle and enhanced contact with people with TB, particularly under overcrowded living conditions. Reduced sun light exposure leads to significantly low skin production of 7DHC and consequently reduced synthesis of D3 metabolites in liver and kidney, resulting in detrimental innate immune response against *M*. *tuberculosis*. Concomitantly, reduced levels of cathelicidin impairs autophagy process. Overall, the combination of these factors would favor a higher susceptibility of being infected.

In the present study, we found that LTBI was more frequent among HHC that were screened in spring, corresponding to TB exposure during winter, which is the season where our contacts had the lowest VD levels of the year. Among possible explanations for this ecological association, it is plausible that in winter there is increased susceptibility to acquire TB infection due to a combination of higher environmental bacterial load exposure as well as an impaired capacity of innate immune system to clear this intracellular bacterium associated to low levels of circulating 25OHD (**Fig 3**). In effect, activation of macrophages via toll-like receptors (TLR) and interferon-gamma are key components of the immune response to *M*. *tuberculosis* that are proposed to be 1,25(OH)D3 dependent [20,21]. In macrophages, 1,25(OH)D3 binds to the vitamin D receptor (VDR) inducing the production of β-defensin 2 and the antimicrobial peptide cathelicidin, that induces autophagy and reversal of phagosome maturation arrest, favoring therefore *M*. *tuberculosis* killing and facilitating antigen processing [22]. Besides, activation of macrophages via TLR leads to upregulation of VDR and upregulation of the enzyme CYP27B1 which converts 25OHD to its active form 1,25(OH)D3. Therefore, under low plasma 25OHD concentrations phagocytosis may be impaired and *M*. *tuberculosis* infection not cleared, adding to the condition of a higher mycobacterial load in the environment due to longer indoor periods, decreased household ventilation, and low UV exposure naturally occurring in winter.

Our findings are concordant with a recent publication from Wingfield *et al*. that also found a significantly higher proportion of HHC having LTBI when screened in spring (in Peru) [27]. The authors hypothesized that intervals from mid-winter peak crowding and trough sunlight accompanied by peaks in VD deficiency are followed by peaks in TB infection in HHC. As the authors state, temporal associations cannot prove causation and many confounding factors may contribute to TB infection seasonality (diet, climate changes, healthcare seeking and access, air quality and other concomitant respiratory-tract infections), however their findings also support that both crowding and VD deficiency are independently associated with TB seasonality [27]. In accordance, other authors, such as Arnedo-Pena *et al*. in Spain have found that low levels of plasma VD associate with positive tuberculin skin test (TST) conversion at follow-up in a small number of contacts [28]. Also, Gibney *et al*. observed that higher VD levels were associated with lower probability of having LTBI in immigrants from Sub-Saharan Africa in Melbourne, Australia [29].

As a known latent TB test limitation, some of our enrolled contacts, particularly migrants from TB endemic countries, may have acquired this infection unsuspectingly in the remote past, even if the presence of LTBI in contacts is usually assumed as a recently acquired infection. However, excluding migrants from the analysis did not modify our findings (data not shown).

Only a few studies have evaluated the effect of VD supplementation in the prevention of LTBI acquisition in contacts. Martineau *et al*. randomly assigned 192 contacts to receive one oral dose of VD (2.5 mg, 100 000 IU) or placebo. A substantial improvement in antimycobacterial innate immunity was observed in the VD versus placebo group, as shown by a growth restriction of recombinant mycobacteria (BCG-lux assay), but not in the acquired immune response parameters [30]. In another promissory proof of concept trial Ganmaa *et al*. explored whether VD supplementation could reduce the risk of TB infection acquisition in school-age children in Mongolia, finding a relative risk of 0.41 (95% CI: 0.16, 1.09) for TST conversion in the group supplemented with VD vs. the placebo group [31].

In addition, we found that VD deficiency was also prominent among patients with recently diagnosed active pulmonary TB, with over 40% of our patients being in the range of severe VD deficiency. Although this finding can reflect a state of ill health or wasting secondary to a chronic infection [32] it may also support the hypothesis that low VD levels confers higher risk of progression from LTBI to active TB [33]. Consistent with our findings, other authors have shown that hypovitaminosis D can be regularly found in subjects with active TB. In Vietnam, Ho-Pham *et al*. found that low serum 25OHD levels (< 30 ng/ml) was a risk of active TB in men, but not in women [34]. In Greenland, a case-control study of TB patients and controls reported that 25OHD levels of < 30 ng/ml or > 56 ng/ml were associated with high risk for active TB [35]. Recently, Arnedo-Pena *et al*. showed in a prospective cohort study a significant inverse association between VD status at baseline and TB incidence at long term follow up (HR 0.88, 95% CI 0.80–0.97), although no further VD measurements were done beyond baseline [33]. In Pakistan, a cohort study of HHC of pulmonary TB patients also found that VD deficiency was a risk factor for developing active TB (RR 5.1 95% CI 1.2–21.3) [36]. Interestingly, a study conducted in London reported a link between VD deficiency with active TB in migrants among all ethnic groups apart from white Europeans and Chinese/South East Asians, proposing a lack of sunlight exposure and a vegetarian diet as main contributors to this deficiency [37]. In contrast, our results show that in Santiago VD deficiency was homogeneously prevalent in patients with active TB and did not differ between migrants and non-migrants at the time of active TB development, nor between subjects from indigenous and non-indigenous origin. We also explored whether hypovitaminosis D could correlate with an increased inflammatory status in plasma—as it has been recently been described for healthy subjects [38]–but, even if our TB patients had elevated inflammatory markers and profound VD deficiency, none of the biomarkers analyzed in plasma (CRP, TNF-α, IL-6) correlated directly with VD levels, what may be explained by variations in the extension and severity of active TB disease.

Differences in the immune modulation of VD may also relate with ethnic variation in VD gene polymorphisms. In Asian populations, a meta-analysis of 23 studies demonstrated different risk of TB development associated with specific VDR polymorphisms with higher risk with the ff genotype of the FokI polymorphism and lower risk with the bb genotype of the BsmI polymorphism [39]. Also, differences in host vitamin D binding protein (DBP) genotype with lower levels of DBP have been described in TB patients with African ancestry versus Eurasian ancestry [40]. There are not studies of VD polymorphisms and TB risk in Chilean population, although in a case-control study conducted in a nearby Peruvian community with a high incidence of TB, VDR TaqI and FokI VDR polymorphisms were not significantly associated with susceptibility to TB [41].

Exposure to cigarette smoke has been associated with VD deficiency in healthy patients, older subjects, and patients with chronic rhinosinusitis [42,43], and a few studies have suggested that cigarette smoke could affect VD metabolism by increasing CYP24A1 (24-hydroxylase, catabolizing enzyme that degrades 1,25(OH)D3) [44] and/or decreasing CYP27B1 (1α-hydroxylase, activating enzyme leading to formation of 1,25(OH)D3) [45]. On the contrary, we found in HHC that tobacco smokers had higher overall levels of VD and lower risk of hypovitaminosis D compared to non-smokers. Many confounding effects could explain this difference, such as an increased outdoor exposure in smokers due to local tobacco regulations (banning indoor smoking) or different dietary habits in smokers [46]. Interestingly, the association of smoking with increased VD levels disappeared after taking season status into account on multivariable analysis.

Not all clinical research has concurred in that VD deficiency confers risk of active TB. A recent study from Owolabi *et al*. conducted in The Gambia surprisingly found not only lower levels of 25OHD in TB contacts than in active TB cases, but also a higher risk of TB progression among contacts having higher levels of VD (median 25OHD 25.0 ng/ml in progressors and 20.3 in non-progressors; p = 0.007) [47]. Despite the small sample size and absence of longitudinal measurements in that study, the results differ strikingly with the majority of previous publications. However–as the authors state–the majority of studies demonstrating insufficient 25OHD in TB patients have been performed in countries with distinct summer and winter seasons and significant differences in levels of natural sunlight, which suggests that there could be geographical differences in the immunological effects of 25OHD.

The results of the present investigation also suggest that migrants (unadjusted analysis) may have a more pronounced VD deficiency than the local population, despite sharing the same urban environment and geographical area. Several factors may explain the findings, such as dietary factors, lower sun exposure habits, skin pigmentation, or variations in genetic metabolism of VD. The largest proportion of migrants came from neighboring countries, Peru and Bolivia, where TB incidence rates are almost 10 times higher (rates of 119 and 117 per 100,000, respectively in year 2015) [1]. Besides carrying higher burden of LTBI, migrants and refugees can be at higher risk of latent TB reactivation and TB transmission within family groups, due to socioeconomic vulnerability, including crowding living conditions, precarious economic conditions preventing them to access well-balanced and nutritious diet, and poor access to health care [48]. In effect, other authors have also found that migrant groups in different geographical areas are more often undernourished and at risk of VD deficiency than native population [49,50].

As differences in socioeconomic conditions and diet inequalities could also be present among all HHC as a complete group, we also explored living conditions, VD levels, and inflammatory biomarkers in a randomly selected small non-HHC control group. Despite its sample size limitation, main findings showed a lower crowding index and a higher plasma VD levels in this non-HHC group. This suggests that HHC compared to TB unexposed population, in the same geographical area, share additional risk factors conferring susceptibility to infection.

Among present study limitations, we did not include a detailed food inquiry, body mass index, or sun exposure habits, therefore we cannot determine the specific underlying cause of VD deficiency in the enrolled subjects. Factors such as darker skin pigmentation can be a risk factor in subjects with a higher proportion of indigenous ancestries, such as in Peruvian and Bolivian migrants [51], however, our multivariable analysis strongly suggests that the most important determinant of VD status is seasonality, not ethnicity nor migrancy status.

In conclusion, the present study shows that VD deficiency is highly prevalent among active TB cases in Chile, in accordance with previous reports from countries with marked sunlight seasonality. Also, we found that the main risk factor for LTBI in HHC was having been tested in spring compared with other seasons of the year. This seasonality for LTBI acquisition risk may relate to winter TB exposure under VD deficiency [27]. The combination of impaired nutritional status contributing to compromised immunity against TB along with crowded household conditions reveals strong health inequities that need improvement. Prospective studies are urgently needed to determine the role of VD supplementation in TB infection prevention, particularly in high-risk communities.

## References

[1] World Health Organization (WHO). WHO | Global tuberculosis report 2016 [Internet]. WHO 2016.

[2] Lonnroth K, Migliori GB, Abubakar I, D’Ambrosio L, de Vries G, Diel R, et al Towards tuberculosis elimination: an action framework for low-incidence countries. Eur Respir J. 2015;45: 928–952. doi: 10.1183/09031936.00214014 25792630 10.1183/09031936.00214014 PMC4391660

[3] Getahun H, Matteelli A, Chaisson RE, Raviglione M. Latent Mycobacterium tuberculosis Infection. N Engl J Med. 2015;372: 2127–2135. doi: 10.1056/NEJMra1405427 26017823 10.1056/NEJMra1405427

[4] Cegielski JP, Arab L, Cornoni-Huntley J. Nutritional risk factors for tuberculosis among adults in the United States, 1971–1992. Am J Epidemiol. 2012;176: 409–22. doi: 10.1093/aje/kws007 22791739 10.1093/aje/kws007 PMC5788452

[5] Bhargava A, Benedetti A, Oxlade O, Pai M, Menzies D. Undernutrition and the incidence of tuberculosis in India: national and subnational estimates of the population-attributable fraction related to undernutrition. Natl Med J India. 27: 128–33. Available: http://www.ncbi.nlm.nih.gov/pubmed/25668081 25668081

[6] Nnoaham KE, Clarke A. Low serum vitamin D levels and tuberculosis: a systematic review and meta-analysis. Int J Epidemiol. 2008;37: 113–9. doi: 10.1093/ije/dym247 18245055 10.1093/ije/dym247

[7] Sita-Lumsden a, Lapthorn G, Swaminathan R, Milburn HJ. Reactivation of tuberculosis and vitamin D deficiency: the contribution of diet and exposure to sunlight. Thorax. 2007;62: 1003–1007. doi: 10.1136/thx.2006.070060 17526677 10.1136/thx.2006.070060 PMC2117124

[8] Zeng J, Wu G, Yang W, Gu X, Liang W, Yao Y, et al A serum vitamin D level <25nmol/l pose high tuberculosis risk: a meta-analysis. PLoS One. Public Library of Science; 2015;10: e0126014 doi: 10.1371/journal.pone.0126014 25938683 10.1371/journal.pone.0126014 PMC4418705

[9] Pareek M, Innes J, Sridhar S, Grass L, Connell D, Woltmann G, et al Vitamin D deficiency and TB disease phenotype. Thorax. 2015; 1–10. 26400877 10.1136/thoraxjnl-2014-206617

[10] Adams JS, Liu PT, Chun R, Modlin RL, Hewison M. Vitamin D in defense of the human immune response. Ann N Y Acad Sci. 2007;1117: 94–105. doi: 10.1196/annals.1402.036 17656563 10.1196/annals.1402.036

[11] Gombart AF. The vitamin D-antimicrobial peptide pathway and its role in protection against infection. Future Microbiol. 2011;4: 1151–1165. 10.2217/fmb.09.87 PMC2821804 19895218

[12] Holick MF, Chen TC. Vitamin D deficiency: a worldwide problem with health consequences. Am J Clin Nutr. 2008;87: 1080S–6S. 18400738 10.1093/ajcn/87.4.1080S

[13] González G, Alvarado JN, Rojas A, Navarrete C, Velásquez CG, Arteaga E. High prevalence of vitamin D deficiency in Chilean healthy postmenopausal women with normal sun exposure: additional evidence for a worldwide concern. Menopause. 2007;14: 455–61. doi: 10.1097/GME.0b013e31802c54c0 17290161 10.1097/GME.0b013e31802c54c0

[14] Koh GCKW, Hawthorne G, Turner AM, Kunst H, Dedicoat M. Tuberculosis Incidence Correlates with Sunshine: An Ecological 28-Year Time Series Study. PLoS One. 2013;8: 1–5. 10.1371/journal.pone.0057752 PMC3590299 23483924

[15] Willis MD, Winston CA, Heilig CM, Cain KP, Walter ND, Mac Kenzie WR. Seasonality of tuberculosis in the United States, 1993–2008. Clin Infect Dis. 2012;54: 1553–60. doi: 10.1093/cid/cis235 22474225 10.1093/cid/cis235 PMC4867465

[16] Martineau AR, Nhamoyebonde S, Oni T, Rangaka MX, Marais S, Bangani N, et al Reciprocal seasonal variation in vitamin D status and tuberculosis notifications in Cape Town, South Africa. Proc Natl Acad Sci U S A. 2011;108: 19013–7. doi: 10.1073/pnas.1111825108 22025704 10.1073/pnas.1111825108 PMC3223428

[17] Guwatudde D, Nakakeeto M, Jones-Lopez EC, Maganda A, Chiunda A, Mugerwa RD, et al Tuberculosis in Household Contacts of Infectious Cases in Kampala, Uganda. Am J Epidemiol. 2003;158: 887–898. 14585767 10.1093/aje/kwg227 PMC2869090

[18] Social CM de D. Encuesta Casen: definiciones e indicadores [Internet]. [cited 6 Apr 2016]. Available: http://observatorio.ministeriodesarrollosocial.gob.cl/casen/casen_def_vivienda.php

[19] Kasai H, Ezaki T, Harayama S. Differentiation of phylogenetically related slowly growing mycobacteria by their gyrB sequences. J Clin Microbiol. 2000;38: 301–8. Available: http://www.pubmedcentral.nih.gov/articlerender.fcgi?artid=88713&tool=pmcentrez&rendertype=abstract 10618105 10.1128/jcm.38.1.301-308.2000 PMC88713

[20] Liu PT, Stenger S, Li H, Wenzel L, Tan BH, Wu K, et al Toll-Like Receptor Triggering of a Vitamin D-Mediate Human Antimicrobial Response. Science. 2006;311: 1770–1773. doi: 10.1126/science.1123933 16497887 10.1126/science.1123933

[21] Fabri M, Stenger S, Shin D-M, Yuk J-M, Liu PT, Realegeno S, et al Vitamin D is required for IFN-gamma-mediated antimicrobial activity of human macrophages. Sci Transl Med. 2011;3: 104ra102 doi: 10.1126/scitranslmed.3003045 21998409 10.1126/scitranslmed.3003045 PMC3269210

[22] Campbell GR, Spector SA. Vitamin D inhibits human immunodeficiency virus type 1 and Mycobacterium tuberculosis infection in macrophages through the induction of autophagy. PLoS Pathog. 2012;8. 10.1371/journal.ppat.1002689 PMC3349755 22589721

[23] Borella E, Nesher G, Israeli E, Shoenfeld Y. Vitamin D: A new anti-infective agent? Ann N Y Acad Sci. 2014;1317: 76–83. doi: 10.1111/nyas.12321 24593793 10.1111/nyas.12321

[24] Shin DM, Yuk JM, Lee HM, Lee SH, Son JW, Harding C V., et al Mycobacterial lipoprotein activates autophagy via TLR2/1/CD14 and a functional vitamin D receptor signalling. Cell Microbiol. 2010;12: 1648–1665. doi: 10.1111/j.1462-5822.2010.01497.x 20560977 10.1111/j.1462-5822.2010.01497.x PMC2970753

[25] Yuk JM, Shin DM, Lee HM, Yang CS, Jin HS, Kim KK, et al Vitamin D3 Induces Autophagy in Human Monocytes/Macrophages via Cathelicidin. Cell Host Microbe. 2009;6: 231–243. doi: 10.1016/j.chom.2009.08.004 19748465 10.1016/j.chom.2009.08.004

[26] Verway M, Bouttier M, Wang TT, Carrier M, Calderon M, An BS, et al Vitamin D Induces Interleukin-1?? Expression: Paracrine Macrophage Epithelial Signaling Controls M. tuberculosis Infection. PLoS Pathog. 2013;9. 10.1371/journal.ppat.1003407 PMC3675149 23762029

[27] Wingfield T, Schumacher SG, Sandhu G, Tovar M a, Zevallos K, Baldwin MR, et al The seasonality of tuberculosis, sunlight, vitamin D and household crowding. J Infect Dis. 2014;210: 1–25. 24596279 10.1093/infdis/jiu121 PMC4130318

[28] Arnedo-Pena A, Juan-Cerdán JV, Romeu-Garcia A, Garcia-Ferrer D, Holguín-Gómez R, Iborra-Millet J, et al Latent tuberculosis infection, tuberculin skin test and vitamin D status in contacts of tuberculosis patients: a cross-sectional and case-control study. BMC Infect Dis. 2011;11: 349 doi: 10.1186/1471-2334-11-349 22171844 10.1186/1471-2334-11-349 PMC3292546

[29] Gibney KB, MacGregor L, Leder K, Torresi J, Marshall C, Ebeling PR, et al Vitamin D deficiency is associated with tuberculosis and latent tuberculosis infection in immigrants from sub-Saharan Africa. Clin Infect Dis. 2008;46: 443–446. doi: 10.1086/525268 18173355 10.1086/525268

[30] Martineau AR, Wilkinson RJ, Wilkinson K a., Newton SM, Kampmann B, Hall BM, et al A single dose of vitamin D enhances immunity to mycobacteria. Am J Respir Crit Care Med. 2007;176: 208–213. doi: 10.1164/rccm.200701-007OC 17463418 10.1164/rccm.200701-007OC

[31] Ganmaa D, Giovannucci E, Bloom BR, Fawzi W, Burr W, Batbaatar D, et al Vitamin D, tuberculin skin test conversion, and latent tuberculosis in Mongolian school-age children: a randomized, double-blind, placebo-controlled feasibility trial. Am J Clin Nutr. 2012;96: 391–6. doi: 10.3945/ajcn.112.034967 22760564 10.3945/ajcn.112.034967 PMC3396446

[32] Autier P, Boniol M, Pizot C, Mullie P. Vitamin D status and ill health: a systematic review. lancet Diabetes Endocrinol. 2014;2: 76–89. 10.1016/S2213-8587(13)70165-7 24622671

[33] Arnedo-Pena A, Juan-Cerdan J V, Romeu-Garcia A, Garcia-Ferrer D, Holguin-Gomez R, Iborra-Millet J, et al Vitamin D status and incidence of tuberculosis among contacts of pulmonary tuberculosis patients. Int J Tuberc Lung Dis. 2015;19: 65–69. doi: 10.5588/ijtld.14.0348 25519792 10.5588/ijtld.14.0348

[34] Ho-Pham LT, Nguyen ND, Nguyen TVTT, Nguyen DH, Bui PK, Nguyen VN, et al Association between vitamin D insufficiency and tuberculosis in a Vietnamese population. BMC Infect Dis. 2010;10: 306 doi: 10.1186/1471-2334-10-306 20973965 10.1186/1471-2334-10-306 PMC2978214

[35] Nielsen NO, Skifte T, Andersson M, Wohlfahrt J, Soborg B, Koch A, et al Both high and low serum vitamin D concentrations are associated with tuberculosis: a case-control study in Greenland. Br J Nutr. 2010;104: 1487–1491. doi: 10.1017/S0007114510002333 20553638 10.1017/S0007114510002333

[36] Talat N, Perry S, Parsonnet J, Dawood G, Hussain R. Vitamin D deficiency and tuberculosis progression. Emerg Infect Dis. 2010;16: 853–855. doi: 10.3201/eid1605.091693 20409383 10.3201/eid1605.091693 PMC2954005

[37] Ustianowski A, Shaffer R, Collin S, Wilkinson RJ, Davidson RN. Prevalence and associations of vitamin D deficiency in foreign-born persons with tuberculosis in London. J Infect. 2005;50: 432–7. doi: 10.1016/j.jinf.2004.07.006 15907552 10.1016/j.jinf.2004.07.006

[38] de Souza WN, Norde MM, Oki É, Rogero MM, Marchioni DML, Fisberg RM, et al Association between 25-hydroxyvitamin D and inflammatory biomarker levels in a cross-sectional population-based study, S??o Paulo, Brazil. Nutr Res. 2016;36: 1–8. doi: 10.1016/j.nutres.2015.10.006 26773775 10.1016/j.nutres.2015.10.006

[39] Gao L, Tao Y, Zhang L, Jin Q. Vitamin D receptor genetic polymorphisms and tuberculosis: updated systematic review and meta-analysis. Int J Tuberc Lung Dis. 2010;14: 15–23. 20003690

[40] Coussens AK, Wilkinson RJ, Nikolayevskyy V, Elkington PT, Hanifa Y, Islam K, et al Ethnic Variation in Inflammatory Profile in Tuberculosis. PLoS Pathog. 2013;9: e1003468 doi: 10.1371/journal.ppat.1003468 23853590 10.1371/journal.ppat.1003468 PMC3701709

[41] Roth DE, Soto G, Arenas F, Bautista CT, Ortiz J, Rodriguez R, et al Association between vitamin D receptor gene polymorphisms and response to treatment of pulmonary tuberculosis. J Infect Dis. 2004;190: 920–7. doi: 10.1086/423212 15295697 10.1086/423212

[42] Jiang CQ, Chan YH, Xu L, Jin YL, Zhu T, Zhang WS, et al Smoking and serum vitamin D in older Chinese people: cross-sectional analysis based on the Guangzhou Biobank Cohort Study. BMJ Open. BMJ Group; 2016;6: e010946 doi: 10.1136/bmjopen-2015-010946 27338881 10.1136/bmjopen-2015-010946 PMC4932269

[43] Mulligan JK, Nagel W, O’Connell BP, Wentzel J, Atkinson C, Schlosser RJ. Cigarette smoke exposure is associated with vitamin D3 deficiencies in patients with chronic rhinosinusitis. J Allergy Clin Immunol. 2014;134: 342–349.e1. doi: 10.1016/j.jaci.2014.01.039 24698317 10.1016/j.jaci.2014.01.039

[44] Matsunawa M, Amano Y, Endo K, Uno S, Sakaki T, Yamada S, et al The aryl hydrocarbon receptor activator benzo[a]pyrene enhances vitamin D3 catabolism in macrophages. Toxicol Sci. 2009;109: 50–58. doi: 10.1093/toxsci/kfp044 19244278 10.1093/toxsci/kfp044

[45] Mulligan JK, Nagel W, O’Connell BP, Wentzel J, Atkinson C, Schlosser RJ. Cigarette smoke exposure is associated with vitamin D3 deficiencies in patients with chronic rhinosinusitis. J Allergy Clin Immunol. 2014;134: 342–349.e1. doi: 10.1016/j.jaci.2014.01.039 24698317 10.1016/j.jaci.2014.01.039

[46] Alkerwi A, Baydarlioglu B, Sauvageot N, Stranges S, Lemmens P, Shivappa N, et al Smoking status is inversely associated with overall diet quality: Findings from the ORISCAV-LUX study. Clinical Nutrition. 2016. 10.1016/j.clnu.2016.08.013 27595637

[47] Owolabi O, Agbla S, Owiafe P, Donkor S, Togun T, Sillah AK, et al Elevated serum 25-hydroxy (OH) vitamin D levels are associated with risk of TB progression in Gambian adults. Tuberculosis. Elsevier Ltd; 2016;98: 86–91. doi: 10.1016/j.tube.2016.02.007 27156622 10.1016/j.tube.2016.02.007 PMC4869593

[48] Huffman S a, Veen J, Hennink MM, McFarland D a. Exploitation, vulnerability to tuberculosis and access to treatment among Uzbek labor migrants in Kazakhstan. Soc Sci Med. 2012;74: 864–72. doi: 10.1016/j.socscimed.2011.07.019 22094009 10.1016/j.socscimed.2011.07.019

[49] Paker-Eichelkraut HS, Bai-Habelski JC, Overzier S, Strathmann S, Heseker H, Stehle P, et al Nutritional status and related factors in elderly nursing home residents: comparative cross-sectional study in migrants and native Germans. J Nutr Gerontol Geriatr. 2013;32: 330–42. doi: 10.1080/21551197.2013.842198 24224940 10.1080/21551197.2013.842198

[50] Ruwanpathirana T, Reid CM, Owen AJ, Fong DPS, Gowda U, Renzaho AMN. Assessment of vitamin D and its association with cardiovascular disease risk factors in an adult migrant population: an audit of patient records at a Community Health Centre in Kensington, Melbourne, Australia. BMC Cardiovasc Disord. 2014;14: 157 doi: 10.1186/1471-2261-14-157 25387481 10.1186/1471-2261-14-157 PMC4233056

[51] Peru population. Demographic data, ethnic groups population and demographics from Peru—CountryReports [Internet]. [cited 25 Dec 2016]. Available: http://www.countryreports.org/country/Peru/population.htm.

